# Awareness and attitudes towards sustainability and climate change amongst students and educators in nursing: A systematic integrative review protocol

**DOI:** 10.1002/nop2.1134

**Published:** 2021-11-23

**Authors:** Jennie Aronsson, Andy Nichols, Paul Warwick, Marie Elf

**Affiliations:** ^1^ School of Nursing and Midwifery Faculty of Health University of Plymouth Plymouth UK; ^2^ Institute of Education Faculty of Arts, Humanities and Business University of Plymouth Plymouth UK; ^3^ School of Health and Welfare Dalarna University Falun Sweden

**Keywords:** climate change, integrative review, nursing education, sustainability

## Abstract

**Aim:**

This review identifies and synthesizes literature related to the awareness of and attitudes towards sustainability and climate change from the perspective of nursing students and educators.

**Design:**

A systematic integrative review.

**Methods:**

The review will follow the five stages outlined by Whittemore and Knafl: problem identification, literature search, data evaluation, data analysis and presentation. The data analysis will be based on inductive content analysis developed by Elo and Kyngäs. Principles of the Cochrane Collaboration and Preferred Reporting Items for Systematic Reviews and Meta Analyses (PRISMA) will also inform the review process.

**Results:**

This review will offer insights about sustainability and climate change in relation to an important target population: the future nursing workforce and those educating its members. Findings might inform curriculum development, potentially contributing to a nursing profession that looks after the health of the planet and the health of the population inhabiting it.

## 1 INTRODUCTION

Climate change is said to be the most urgent public health threat of the 21st century (Costello et al., 2009). Risks to health include air pollution, forced migration and changing patterns of infectious disease, compromising physical health and mental wellbeing; effects that are more likely to impact on vulnerable populations (Solomon & LaRocque, 2019; Watts et al., 2018). In 2015, the United Nations produced the Sustainable Development Goals as a call for action to end poverty, protect the planet and establish peace and prosperity for all (United Nations, 2015). The International Council of Nursing (2018) has called for nurses to act as leaders in building climate resilient health systems by, for example adopting sustainable healthcare practices, engaging in health and climate research and developing climate‐informed health programmes. Yet delivering healthcare contributes to climate change through greenhouse gas emissions, the use of toxic materials and the production of vast amounts of waste (Griggs et al., 2017; Richardson et al., 2016).

Anåker and Elf (2014) argue that awareness of sustainability and the consequences of unsustainable development will prepare nurses for future challenges. Sustainability in nursing is defined in this work in accordance with their concept analysis, which concluded that:

The concept of sustainability in nursing can be defined from a core of knowledge in which ecology, global and holistic comprise the foundation. The use of the concept of sustainability includes environmental considerations at all levels. The implementation of sustainability will contribute to a development that maintains an environment that does not harm current and future generation’s opportunities for good health. Anåker & Elf (2014, p.387)

Cruz et al. (2018) suggest that increased awareness about climate change and sustainability can motivate nurses to adopt sustainable practices; this includes waste management in the clinical environment (Nichols & Mukonoweshuro, 2017). Aronsson et al. (2020) found that educational sessions that focused on the relevance of climate change and sustainability to health and health care in undergraduate courses could lead nursing students to challenge unsustainable clinical practice. However, barriers to doing so included a resistance to change amongst clinical staff, in some cases related to negative staff attitudes (Aronsson et al., 2020). To learn about climate change and sustainability, Cruz et al. (2018) suggest that nursing students need positive attitudes towards these concepts, including a sense of responsibility, a willingness to change and a confidence in the future (Lopez‐Medina et al., 2019). To prepare future nurses for their role, nursing educators have a duty to ensure that nursing programmes reflect changing needs, developments, priorities and expectations in health and health care. Goodman (2011) argues that a curriculum that focuses on developing biomedical skills rather than addressing global health and sustainability will not equip nursing students for future challenges that will face the population as a result of anthropogenic climate change.

Arguably, having an awareness of climate change, accompanied by a positive attitude towards sustainability, are important aspects of nursing and, therefore, nursing education. In view of this, this integrative review aims to explore the awareness of and attitudes towards sustainability and climate change from the perspective of nursing students, and from nursing educators.

### 1.1 Aim

This integrative review aimed to identify research on the awareness of and attitudes towards sustainability and climate change from the perspective of nursing students and educators.

## 2 THE STUDY

### 2.1 Design

We will undertake an integrative review of the literature to analyse and synthesize results from studies (Whittemore & Knafl, 2005). This design is suitable when the researcher is expecting heterogeneous literature and enables a comprehensive understanding of the phenomena around climate changes and sustainability. The review will follow the five stages outlined by Whittemore and Knafl (2005): (1) problem identification, (2) literature search, (3) data evaluation, (4) data analysis and (5) presentation. The principles of the Cochrane Collaboration (2013) and Preferred Reporting Items for Systematic Reviews and Meta Analyses (PRISMA) (Page et al., 2021) will also inform the review process.

### 2.2 Methods

#### 2.2.1 Problem identification

The Sample, Phenomenon of Interest, Design, Evaluation and Research type (SPIDER) framework (Cooke et al., 2012) will be used to formulate the research question and determine the inclusion and exclusion criteria, which are outlined in Table 1.

**TABLE 1:** Inclusion and exclusion criteria

	Inclusion	Exclusion
Sample	Studies focusing on student nurses, at any point of their education. Studies focusing on academic and/or clinical nurse educators, regardless of the profession of the clinical nurse educator. Studies from any country worldwide.	Studies focusing on perspectives of healthcare professionals not involved in nursing education
Phenomenon of Interest	Studies focusing on the concepts sustainability and/or climate change, or any search terms related to these.	Studies where the definition of “sustainability” does not align with the definition used in this review: “The concept of sustainability in nursing can be defined from a core of knowledge in which ecology, global and holistic comprise the foundation. The use of the concept of sustainability includes environmental considerations at all levels. The implementation of sustainability will contribute to a development that maintains an environment that does not harm current and future generation's opportunities for good health.” (Anåker & Elf, 2014 p. 387)
Design	Empirical research studies using any research design Published journal articles reporting on completed studies	Theoretical/conceptual studies and opinion papers Protocols and grey literature
Evaluation	Studies exploring the awareness of the phenomena of interest from the perspective of the target sample, in relation to their professional/student role or their role as private persons/global citizens Studies exploring the attitudes towards the phenomenon of interest from the perspective of the target sample, in relation to their professional/student role or their role as private persons/global citizens	
Research type	Primary research using a qualitative, quantitative or a mixed methods approach.	Secondary research will be excluded; however, the reference lists of any literature reviews (of any design) retrieved in the search will be manually searched for primary studies to include.

Studies will be included regardless of year of publication. For studies written in other languages in English, an attempt will be made to translate these to be able to include them if they meet the inclusion criteria.

#### 2.2.2 Literature search

The CINAHL, MEDLINE, EMBASE, Web of Science, British Education Index, GreenFILE and Scopus electronic databases will be searched. These data bases have been chosen in consultation with an Information Specialist to ensure inclusion of a broad range of literature pertaining to research in the fields of health care, social sciences, higher education and climate change. The search terms have been selected in discussion in the review team and are listed in Appendix 1.

The search results from each database search will be imported into EndNote and transferred to Rayyan (https://rayyan.qcri.org/welcome) where the screening will take place based on inclusion and exclusion criteria listed in Table 1. Duplicate documents will be removed from the total list of citations obtained. Initially, 10% of studies will be screened independently by three members of the review team, based on title and abstract. This process will ensure interrater reliability, thus establishing an unbiased and consistent data collection strategy (Polit & Beck, 2018). Following this initial phase of the screening, two members of the review team will screen all studies, based on title and abstract. Any studies included at this stage will then be subject to screening by full‐text articles by the same two members of the review team.

As a second search strategy, reference lists of included articles will be manually reviewed. In addition to this, relevant literature reviews (of any design) that were retrieved at the initial screening phase will be manually reviewed for primary studies that might meet the inclusion criteria.

Disagreements between reviewers in the abstract or full‐text screening phase will be resolved through discussion until an agreement is reached. If necessary, a third reviewer will be used to resolve any discrepancies as a measure of investigator triangulation, whereby decisions are made collaboratively to reduce biased decisions (Polit & Beck, 2018).

All reviewers will inspect the final list of included studies, before progressing to the next stage of the review. A PRISMA flow diagram (Page et al., 2021) will be created to illustrate the completed search.

#### 2.2.3 Data evaluation

All included articles will be critically appraised independently by two members of the review team, using the Mixed Methods Appraisal Tool (MMAT, Hong et al., 2018), which allows for studies of different designs (qualitative and quantitative) to be appraised. The quality assessment for each study will be compared and discussed, and any discrepancies will be discussed collaboratively in the research team, again drawing on investigator triangulation to ensure trustworthiness at this important stage of the integrative review (Polit & Beck, 2018). The quality of each study will be clearly presented in the integrative review to give an indication of the overall strength of the body of evidence.

#### 2.2.4 Analysis

A data extraction Excel spreadsheet based on Cochranes framework (Cochrane Library, 2013) will be developed. From each included study, data about the awareness of, and/or attitudes towards, sustainability and climate change will be extracted, together with relevant study characteristics, by one member of the research team, with another team member independently extracting data from 10% of studies, randomly selected, to ensure interrater reliability (Polit & Beck, 2018).

A narrative synthesis will be performed to explain the findings based on inductive content analysis developed by Elo & Kyngäs (2008). This data analysis method aligns with the overarching constant comparison method proposed by Whittemore and Knafl (2005) and has been used previously in integrative reviews (Kivimaki et al., 2019).

#### 2.2.5 Presentation

Findings will be presented under the headings of main categories from the data analysis, with examples from primary sources to ensure authenticity (Elo & Kyngäs, 2008; Polit & Beck, 2018).

The study will be reported in accordance with the PRISMA guidelines (Page et al., 2021).

### 2.3 Ethics

Research Ethics Committee approval or patient consent will not be required as this is a protocol for an integrative review of published papers.

## 3 DISCUSSION

This integrative review will seek to comprehensively synthesize the existing body of research related to the awareness of and attitudes towards sustainability and climate change from the perspective of nursing and educators. The findings of the review could be considered when planning nursing curricula in relation to intended learning outcomes and pedagogies. Although some higher education institutes across Europe and elsewhere are already incorporating sustainability in nursing education (Richardson et al., 2016), Lopez‐Medina et al. (2019) note that knowledge about sustainability and climate change principles is largely absent from nursing curricula. More evidence in this field would highlight the importance of these topics to nursing, potentially encouraging a universal incorporation of sustainability education in nursing curricula.

Munn et al. (2018) highlights the importance of translating research evidence into practice, so that relevant people are aware of, have access to and understand the most up‐to‐date information. Additionally, research projects are time consuming and costly; thus, there is an ethical argument to disseminate findings for the benefit of patients/health care/wider society (Williamson & Whittaker, 2020). The completed integrative review will be published in a relevant journal aimed at nursing educators in academia and practice, to facilitate knowledge transfer. Climate change and sustainability are topics that are increasingly highlighted in the news, and there is an increasing awareness of these issues in society; however, this integrative review offers insight into these concepts in relation to an important target population: the future nursing workforce, and those who educate them (and, therefore, have an impact upon their future practice).

### 3.1 Limitations

There are some potential limitations in the study design. The integrative review may not identify all relevant studies, because of difficulties in defining the concepts awareness and attitudes. To overcome this, we have identified a wide range of synonyms as search terms, to include as many definitions as possible (Appendix 1). Additionally, as society evolves, definitions of concepts change over time; therefore, by not restricting the search to certain dates, there is a risk of missing older studies where the terminology used might have been different. However, sustainability and climate change are concepts that are increasingly highlighted in the literature as important aspects of health care and nursing education; hence, it is expected that most studies found through this review will be published in the last ten years.

The study will include quantitative and qualitative studies and few critical appraisal tools for mixed study reviews are available. We chose MMAT since it has shown content validity and reliability (Crowe & Sheppard, 2011). However, results have also shown mixed psychometric outcomes (Souto et al., 2015). Thus, in this review, we will have consensus discussion after independent appraisal to minimize bias.

There is no specific reporting guideline for integrative or mixed‐method reviews. The use of review methods and a clear report of decision‐making will ensure a transparent review process. The use of the PRISMA checklist for systematic reviews (Page et al., 2021) will give a systematic process for reporting the review of evidence and enhance reliability.

We will adopt a narrative synthesizing of data. The interpretation of data may be open to bias but the involvement of the whole review team, with multiple perspectives during the entire process, will limit the risk of bias.

### 3.2 Conclusion

The findings of this integrative review might inform curriculum development, potentially contributing to a future nursing workforce made up of responsible global citizens who look after the health of the planet, and the health of the population inhabiting it.

## CONFLICT OF INTEREST

None declared.

## AUTHOR CONTRIBUTIONS

Jennie Aronsson made substantial contributions to conception and design of the study. She was involved in drafting the manuscript, revising it critically for important intellectual content and giving final approval of the version to be published. She is accountable for all aspects of the work in ensuring that questions related to the accuracy or integrity of any part of the work are appropriately investigated and resolved. Andy Nichols has made contributions to the conception and design and has been involved in drafting the manuscript and given final approval of the version to be published. Paul Warwick has made contributions to the design of the study and been involved in the drafting of the manuscript and given final approval of the version to be published. Marie Elf made contributions to conception and design of the study. She has been involved in drafting the manuscript and revising it critically for important intellectual content and given final approval of the version to be published.

## Data Availability

Data sharing is not applicable to this article as no new data were created or analysed in this protocol.

## APPENDIX 1. Search terms

**Table:**

Sample AND Phenomenon of Interest AND Evaluation		
“student nurse*” OR “nursing student*” OR “nurse* educator*” OR “nurse* lecturer*” OR “nurse* mentor*” OR “nurse* teacher*” OR “nurse* tutor*” OR “practice assessor” OR “practice supervisor”	sustainable* OR “climate* change” OR “global warming” OR “greenhouse effect” OR “climate crisis” OR “climate emergency” OR “climate action” OR “environmental change” OR “energy resilience” OR “carbon footprint” OR “carbon reduction” OR “carbon removal” OR “zero carbon” OR “planetary health”	awareness OR knowledge OR understanding OR cognition OR attitude* OR view* OR opinion* OR perception* OR perspective* OR feeling* OR value* OR belief* OR thought* OR “behaviour*r change” OR *action OR response
